# Specific increase of *Fusobacterium* in the faecal microbiota of neonatal calves infected with *Cryptosporidium parvum*

**DOI:** 10.1038/s41598-019-48969-6

**Published:** 2019-08-29

**Authors:** Madoka Ichikawa-Seki, Daisuke Motooka, Aiko Kinami, Fumi Murakoshi, Yoko Takahashi, Junya Aita, Kei Hayashi, Atsushi Tashibu, Shota Nakamura, Tetsuya Iida, Toshihiro Horii, Yoshifumi Nishikawa

**Affiliations:** 1 Laboratory of Veterinary Parasitology, Faculty of Agriculture, Iwate University, 3-18-8 Ueda, Morioka, 020-8550 Japan; 2 Department of Infection Metagenomics, Genome Information Research Center, Research Institute for Microbial Diseases, Osaka University, Suita, 565-0871 Japan; 3 Famille202, 110-16, Ogama-todate, Takizawa, Iwate 020-0762 Japan; 4 National Research Center for Protozoan Diseases, Obihiro University of Agriculture and Veterinary Medicine, Nishi 2-13 Inada-cho, Obihiro, 080-8555 Japan; 5 Department of Infectious Diseases, Kyoto Prefectural School of Medicine, 465, Kajiicho, Kawaramachi-hirokoji, Kamigyo-ku, Kyoto, 602-8566 Japan; 6 Tyubu Area Center Veterinary Clinic, Iwate Agricultural Mutual Aid Association, 821 Shimoneko, Hanamaki, Iwate 025-0025 Japan; 7 Laboratory of Veterinary Parasitology, Faculty of Veterinary Medicine, Okayama University of Science, 1-3 Ikoinooka, Imabari, 794-8555 Japan

**Keywords:** Metagenomics, Parasite host response

## Abstract

The faecal microbiota plays a critical role in host health, with alterations in the human faecal microbial composition associated with various conditions, particularly diarrhoeal diseases. However, little is known about microbial changes during cryptosporidiosis, one of the most important diarrhoeal diseases caused by protozoa in cattle. In this study, alterations in the faecal microbiota of neonatal calves as a result of *Cryptosporidium parvum* infection were investigated on a *C. parvum*-positive farm. Comparisons were made among groups of *C. parvum-*infected, rotavirus-infected, and the pathogen-negative calves. A specific increase in the abundance of *Fusobacterium* was observed in the faecal microbiota of *C. parvum*-infected animals. Diarrhoea severity increased in accordance with the abundance of *C. parvum* and *Fusobacterium*. Moreover, the specific increase of *Fusobacterium* appeared to be a universal feature of *C. parvum* infection, since neonatal calves from geographically separated areas showed the same result. These observations indicated that the growth of *Fusobacterium* may be an important aggravating factor of cryptosporidiosis.

## Introduction

The gut microbiota plays a critical role in the gut health of the host. It protects against enteropathogens, extracts nutrients and energy from food and contributes to normal immune function. Disruption of the normal composition of the gut microbiota has been associated with obesity, malnutrition, inflammatory bowel disease, neurological disorders and several cancers^1^. Alterations of the normal human gut microbiota have been documented, particularly in diarrhoeal diseases such as antibiotic-associated diarrhoea^2^, inflammatory bowel disease^3^, acute post radiotherapy diarrhoea^4^ and irritable bowel syndrome^5,6^.

In a previous study of the faecal microbiota of neonatal calves, Firmicutes was the most abundant phylum, with a prevalence ranging from 63.84–81.90%, followed by Bacteroidetes (8.36–23.93%), Proteobacteria (3.72–9.75%), Fusobacteria (0.76–5.67%) and Actinobacteria (1.02–2.35%)^7^. Changes in the digestive tract microbiome have also been identified in cattle exhibiting diarrhoea^8,9^. While a shift in the faecal microbiota of cattle infected with *Mycobacterium avium* subsp. *paratuberculosis* (Johne’s disease) has been reported^10^, limited information is available about the effects of other infectious diarrhoeal pathogens on the faecal microbiota of cattle.

*Cryptosporidium parvum* is a coccidian protozoan parasite that causes enteric infection and diarrhoeal disease in many mammals, including both immunocompetent and immunocompromised humans^11^. The parasite is widely distributed and is a common cause of severe neonatal diarrhoea among calves, with cryptosporidiosis being one of the most important infectious diarrhoeal diseases caused by protozoa for the cattle industry. While cryptosporidiosis is usually mild and self-limiting in immunocompetent humans, individuals with various immune disorders, including acquired immune deficiency syndrome, often contract chronic, life-threating infections^12^. While nitazoxanide has been licensed for the treatment of *Cryptosporidium*-induced diarrhoea in humans^13^, its efficacy in calves remains unclear^14^. Although one study reported beneficial effects of nitazoxanide in the treatment of cryptosporidiosis in experimentally-infected neonatal calves^15^, another study revealed no prophylactic or therapeutic efficacy^16^. Therefore, clinical disease control in calves has been hampered by the lack of drugs and vaccines that are effective for either treatment or prevention of cryptosporidiosis^17,18^.

The gut microbiota is thought to affect resistance to *C. parvum* infection because germfree adult immunocompetent mice showed high susceptibility to infection^19^. Moreover, a study using severe combined immunodeficient (SCID) mice supported the hypothesis that resistance of adult mice to *C. parvum* infection does not require a specific immune response but can be mediated by nonspecific mechanisms associated with the presence of intestinal microflora^20^. This hypothesis was based on results showing that *C. parvum* was not readily detected in flora-bearing adult SCID mice, while germfree SCID mice were heavily infected following challenge with the parasite^20^. These observations indicate that some interaction occurs between *C. parvum* and the faecal microbiota in infected hosts.

Administration of live *Lactobacillus* bacterial cell-free supernatants reduces the viability of *C. parvum* oocysts *in vitro*^21,22^. Furthermore, probiotics can limit *C. parvum* infection in immunocompromised individuals in mouse models of the disease^23,24^, with similar results observed for human cases^25^. However, probiotic treatment of calves infected with *C. parvum* did not result in a significant decrease in the incidence of diarrhoea or oocyst shedding compared with the controls^26^.

At present, nothing is known about the faecal microbiota of neonatal calves infected with *C. parvum*. Interactions between the faecal microbiota and *C. parvum* must be analysed to understand pathophysiological changes that occur during disease progression. To address this knowledge gap, we examined the faecal microbiota profiles of neonatal calves from a *C. parvum*-endemic farm. Metagenomic sequencing analysis was conducted using the IonPGM, and the composition of the microbiota revealed by the high-throughput sequencing was re-examined and confirmed by quantitative polymerase chain reaction (qPCR) analysis. Moreover, neonatal calves from different regions of Japan were examined to evaluate the universality of observed alterations in the faecal microbiota caused by *C. parvum* infection.

## Results

### Specific increase of *Fusobacterium* abundance in *C. parvum*-only infected calves revealed by metagenomic analysis

Metagenomic analysis based on 16 S rRNA gene sequences was performed for a total of 120 faecal samples collected at six time points from 20 neonatal Holstein calves (Table S1). The calves were aged between 0 and 15 days old and were located on a farm (farm #A) in Iwate Prefecture, Japan. All of the calves were female and were born in identical cattle sheds. Birth weights ranged from 35–45 kg. All treatments after birth, including colostrum practices, were identical for all calves.

**Figure 1:**
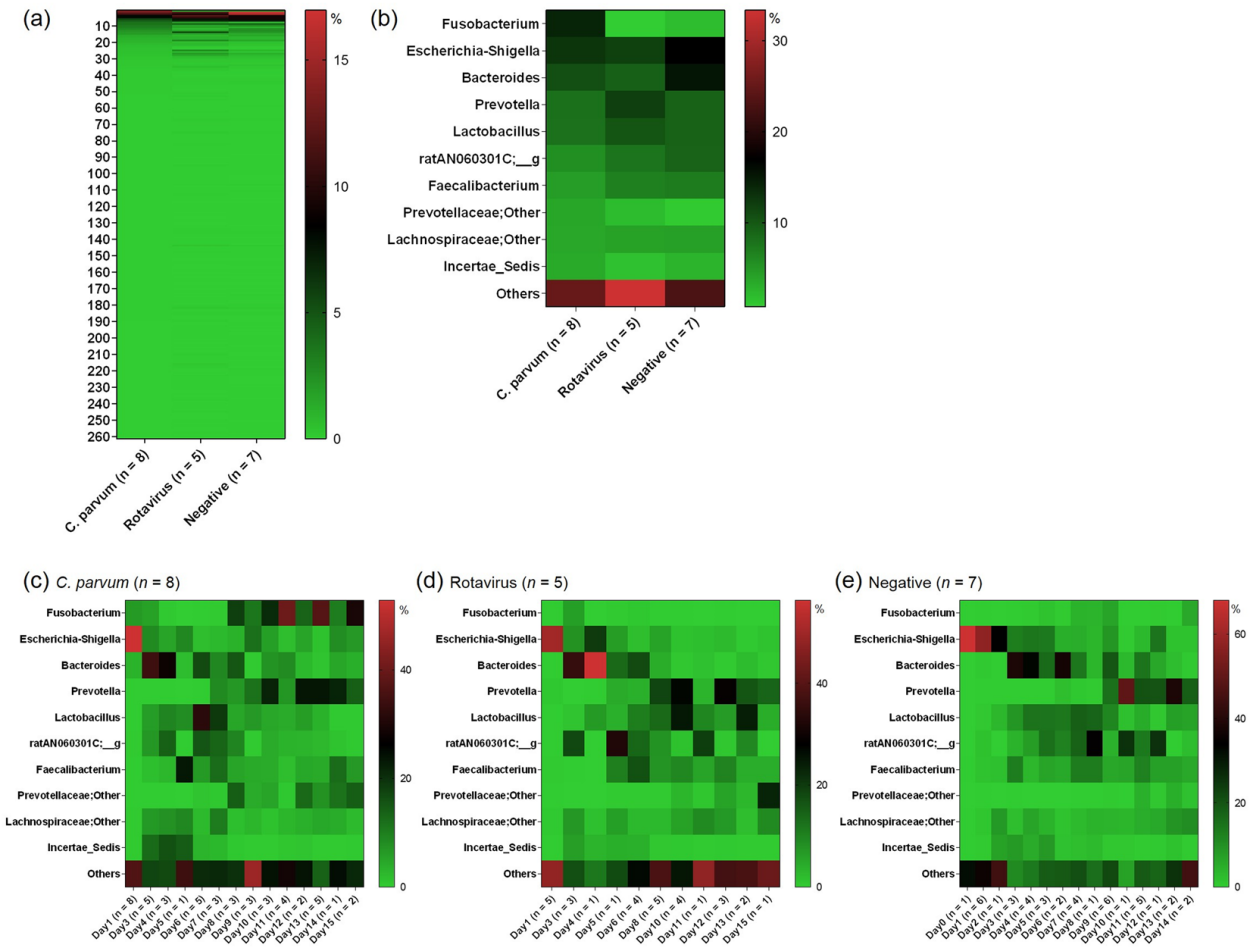
Distribution of bacterial relative abundance among the three groups of faecal samples collected at farm #A: *Cryptosporidium parvum*-only-infected (*n* = 8), rotavirus-only-infected (*n* = 5) and pathogen-negative (*n* = 7). An average value for the relative abundance across the six sampling points for each genus is shown using a gradient scale. (**a**) The 261 analysed genera (Table S2) were plotted as rows in which the most abundant genus in the *C. parvum*-only infected group is shown at the top. The next most abundant genera are shown sequentially in order of abundance. (**b**) The 10 most abundant genera (shown on the left-hand side) in the *C. parvum*-only-infected group in comparison with the corresponding abundances in the rotavirus-only-infected and pathogen-negative groups. The top 10 genera were obtained from (**a**). A specific increase of *Fusobacterium* in the *C. parvum*-only-infected group was suggested. (**c**–**e**) Time-dependent change of bacterial relative abundance among *C. parvum*-only-infected, rotavirus-only-infected and pathogen-negative groups. The column titles are the numbers of faecal samples collected in each age in days (see Table S1).

The distribution of the relative abundance of the different bacterial genera is shown in Fig. 1a for each of the three groups: *C. parvum*-only-infected (*n* = 8), rotavirus-only-infected (*n* = 5) and the pathogen-negative group (*n* = 7) determined by the commercial immunochromatographic test (ICT) strips (Bio-X Diagnostics SPRL, Jemelle, Belgium). None of the animals tested positive for coronavirus infection.

The most abundant genus in *C. parvum*-only-infected calves was *Fusobacterium* (14.1%, average of all samples), followed by *Escherichia-Shigella* (12.8%). The relative ratio of *Fusobacterium* was low in the rotavirus-only-infected (0.7%) and pathogen-negative (2.0%) groups, while the relative ratios of *Escherichia-Shigella* in the two groups (11.9% and 17.0%, respectively) were similar to that observed in the *C. parvum*-infected group (Fig. 1b, Table S2).

**Figure 2:**
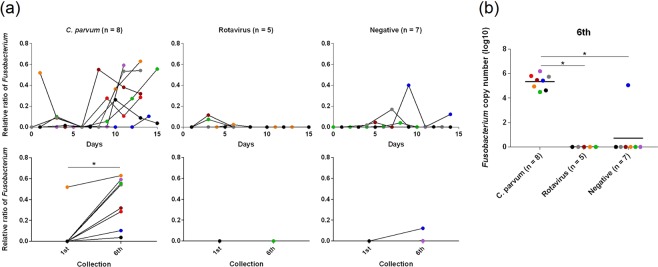
Comparisons of *Fusobacterium* loads obtained by metagenomic analysis among the three groups: *Cryptosporidium parvum*-only-infected (*n* = 8), rotavirus-only-infected (*n* = 5), and the pathogen-negative (*n* = 7) samples collected from farm #A. (**a**) The relative ratios of *Fusobacterium* for all the calves in relation to their age (upper). Wilcoxon test was performed to reveal the significant differences between the 1^st^ (0–1 day old) and the 6^th^ (10–15 days old) sampling points in the three groups (lower). The increase of *Fusobacterium* was detected in *C. parvum*-only-infected group (*P < 0.05). (**b**) The specific increase in the abundance of *Fusobacterium* in *C. parvum*-only-infected calves at the 6^th^ sampling point was confirmed by quantitative PCR (Kruskal-Wallis test, *P < 0.05).

The abundance of *Fusobacterium* appeared to be increased in the faecal microbiota of *C. parvum*-only-infected calves (Figs 1c and 2a). A significant increase was found in the abundance of the bacteria between the 1^st^ (0–1 day old) and the 6^th^ (10–15 days old) sampling points (Fig. 2a, Wilcoxon test, *P* < 0.05). No significant increase was detected in rotavirus-only-infected (*n* = 5) and the pathogen-negative calves (*n* = 7) (Fig. 2a). No other genus-specific increase was observed among the top 10 genera identified in the *C. parvum*-only-infected samples (listed in Fig. 1b–e).

The results of the metagenomic analysis were reproduced by the qPCR assays at the 6^th^ sampling point (10–15 days old) (Fig. 2b). A specific increase in the abundance of *Fusobacterium* was detected in *C. parvum*-only-infected calves (*n* = 8) in comparison with the rotavirus-only-infected (*n* = 5) and the pathogen-negative calves (*n* = 7). A significant difference was observed between the *C. parvum*-positive group and the other groups (Kruskal-Wallis test, *P* < 0.05).

### Abundance of *C. parvum* and *Fusobacterium* in the samples from other locations as determined by qPCR

**Figure 3:**
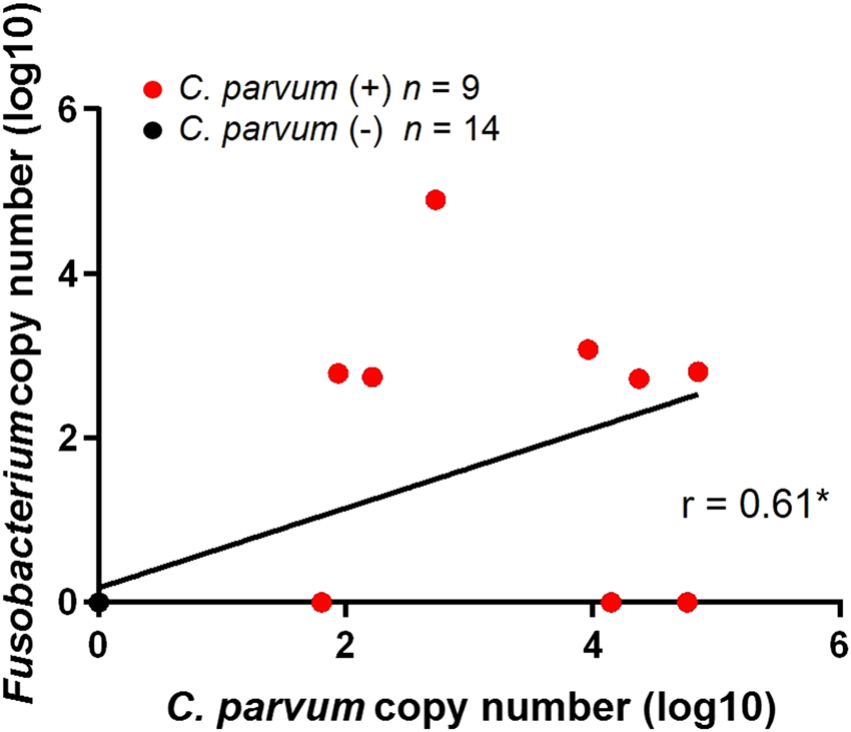
*Fusobacterium* loads in samples from *Cryptosporidium parvum-*positive or -negative calves aged 11–15 days from the farms in Okinawa (*n* = 7), Kagoshima (*n* = 1), Iwate (*n* = 9) and Hokkaido (*n* = 6) prefectures. The increase in abundance of *Fusobacterium* in *C. parvum*-positive samples (*n* = 9) was detected in six samples by quantitative PCR (qPCR). No increase was observed in *C. parvum*-negative samples (*n* = 14). A highly significant correlation was observed (r = 0.61, *P < 0.05).

An increase in the abundance of *Fusobacterium* was also detected in 11–15 days old *C. parvum-*positive calves from the farms located in Okinawa (*n* = 7), Kagoshima (*n* = 1), Iwate (*n* = 9) and Hokkaido (*n* = 6) prefectures (Supplementary Fig. S1). The ages of these calves were very similar to those of calves from farm #A at the 6^th^ sampling point (10–15 days old). The increase in abundance of *Fusobacterium* was only observed in *C. parvum*-positive calves (*n* = 9), with no increase detected in the *C. parvum*-negative calves (*n* = 14) (Fig. 3). Moreover, the *Fusobacterium* load showed a strong positive correlation with that of *C. parvum* (r = 0.61, *P* < 0.05) (Fig. 3).

### Association between the presence of *C. parvum* and *Fusobacterium* in faecal samples from the different locations

Fisher’s exact test revealed that the number of *Fusobacterium*-positive/*C. parvum*-positive samples was significantly greater than the number of *Fusobacterium*-positive/*C. parvum*-negative samples from the different locations (Okinawa, Kagoshima, Iwate and Hokkaido prefectures) (*P* < 0.05) (Table 1).

**Table 1:** Association between the presence of *Cryptosporidium parvum* and *Fusobacterium* in faecal samples collected from 11–15 days old calves in the different locations.

	*Fusobacterium* positive		*Fusobacterium* negative		total
*C. parvum* positive	6*	(26.1%)	3	(13.0%)	9
*C. parvum* negative	0	(0%)	14*	(60.9%)	14
total	6		17		23

Statistically significant associations were observed between *Cryptosporidium parvum* and *Fusobacterium* (Fisher’s exact test, *P < 0.05).

### Oocyst numbers and faecal scores during *C. parvum* infections in relation to the relative ratios of *Fusobacterium*

**Figure 4:**
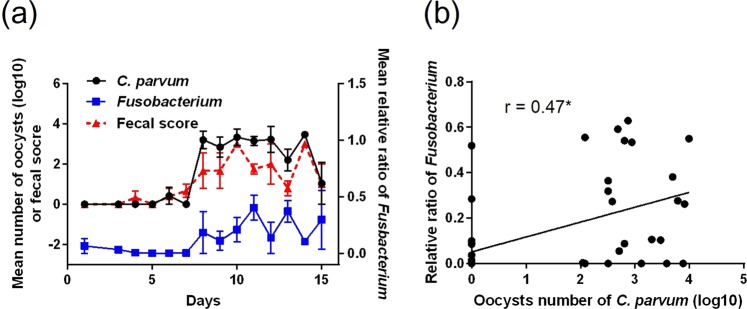
Oocyst numbers and faecal scores during *Cryptosporidium parvum* infections in relation to the relative ratios of *Fusobacterium*. (**a**) Average numbers of *C. parvum* oocysts, relative ratios of *Fusobacterium*, and faecal scores for each sampling day for the *C. parvum*-positive samples from farm #A. (**b**) Correlation between the number of *C. parvum* oocysts and the relative ratios of *Fusobacterium* for the *C. parvum*-positive samples from farm #A. A moderately positive correlation was observed (r = 0.47, *P < 0.05).

The number of *C. parvum* oocysts detected in the infected neonatal calves from farm #A increased at day 8 (Fig. 4a). Again, the relative ratios of *Fusobacterium* began to increase at the same time point (Fig. 4a). The faecal scores, indicating severity of diarrhoea, increased in accordance with the abundance of *C. parvum* and *Fusobacterium* (Fig. 4a). A moderately positive correlation was observed between the number of oocysts and the relative ratio of *Fusobacterium* (r = 0.47, *P* < 0.05) (Fig. 4b).

## Discussion

The metagenomic analysis conducted in this study revealed that the abundance of *Fusobacterium* was particularly increased in *C. parvum*-only-infected calves (Figs 1 and 2a). Although the *Fusobacterium* species detected in this study was not identified, the specific increase in *Fusobacterium* among *C. parvum*-positive samples from farm #A was confirmed by qPCR analysis (Fig. 2b).

*Fusobacterium* species are anaerobic, elongated, Gram-negative rods. While there are multiple species of *Fusobacterium*, the species most commonly associated with human and animal disease is *F. necrophorum*, an opportunistic pathogen that causes numerous necrotic conditions (necrobacillosis) and both specific and non-specific infections in a variety of animals. Bovine liver abscesses and foot rot caused by *F. necrophorum* are significant concerns in the cattle industry^27^.

How factors such as the environment and diet shape the human faecal microflora remains unclear. Nonetheless, studies have revealed increased levels of *Prevotella* or *Bacteroides* in response to a high-fibre diet or a long-term diet rich in animal proteins, respectively^1^. In calves, probiotic administration or the use of lactic acid bacteria has been identified as a tool to maintain the intestinal microbial balance and to prevent the establishment of opportunistic pathogens^28^. Therefore, dietary differences between farms, especially in the presence or absence of probiotic supplementation, will likely affect the composition of the faecal microbiota of calves. However, in this study, the specific increase of *Fusobacterium* was observed not only in animals from farm #A, but also in calves from completely different locations (Table 1, Fig. 3). This suggests that the growth of *Fusobacterium* is a common feature among *C. parvum*-positive calves, regardless of differences in diet and environmental conditions between farms.

The oocyst shedding pattern of *C. parvum* observed in this study (Fig. 4a) was similar to those recorded in previous reports^29,30^. Interestingly, the initiation of oocyst shedding (at 8 days old) appeared to coincided with the increase of *Fusobacterium* (at 8 days old) (Fig. 4a). The protozoan infection occurred prior to the increase in *Fusobacterium* growth because the oocyst incubation period in dairy cattle is 3 to 6 days prior to shedding^11^. Moreover, the relative ratios of the bacteria tended to increase in accordance with increasing numbers of oocysts (Fig. 4b). These observations suggest that the increase in *C. parvum* may benefit the growth of *Fusobacterium*. The beneficial interaction between these two microbes appeared to be universal based on the strong positive correlation between *C. parvum* and *Fusobacterium* observed in faecal samples from different origins (Fig. 3).

Previous studies have revealed that cryptosporidiosis symptoms are strongly correlated with the load of *C. parvum* in host faecal samples. Challenge of human volunteers with *C. parvum* showed that the number of oocysts excreted in faeces was significantly higher in subjects with diarrhoea than in those with enteric symptoms but no diarrhoea^31^. In addition, a previous study on risk factors for neonatal calf diarrhoea caused by *C. parvum* reported a significant association between high oocyst number and the occurrence of diarrhoea^32^. In the present study, faecal scores appeared to worsen (Fig. 4a) with corresponding increases in the abundance of both *C. parvum* (Fig. 4a) and *Fusobacterium* (Figs 2a and 4a). Therefore, the increase of *Fusobacterium* appears to be an important aggravating factor for cryptosporidiosis because the growth of the bacterium increases the *C. parvum* load in neonatal calves (Figs 3b and 4b). An animal model using neonatal calves for *C. parvum* challenge will be required in the future to gather direct evidence of the co-increase of *C. parvum* and *Fusobacterium*.

We propose that the specific increase in *Fusobacterium* may be caused by the following mechanism. Damage to microvilli on the surface of intestinal epithelial cells caused by the growth of *C. parvum* increases susceptibility to *Fusobacterium* infection. *Fusobacterium* then becomes a dominant genus and grows effectively because of its ability to adhere to and invade intestinal cells^33–35^, which may cause severe diarrhoea in cattle. Because this hypothesis remains unconfirmed, a pathological analysis of intestinal cells is needed to understand the beneficial interaction between *C. parvum* and *Fusobacterium*.

Clinical studies on human diarrhoeal diseases^35,36^ have shown that *Fusobacterium* is found in colonic tissues of patients with inflammatory bowel disease. *Fusobacterium varium* is a known causative agent of ulcerative colitis^37^. A previous study showed that a 2-week course of combination antibiotic therapy (amoxicillin, 1,500 mg; tetracycline, 1,500 mg; and metronidazole, 750 mg/day) reduces the density of *F. varium* in the mucosa, resulting in a higher remission rate in the treatment group compared with the control group^38^. The present study suggests that this kind of antibiotic therapy targeting *Fusobacterium* may also relieve the severity of diarrhoea in cryptosporidiosis cases.

This is the first study to reveal a significant correlation between *Fusobacterium* and *C. parvum* in cases of neonatal calf diarrhoea. The increase of *Fusobacterium* in the faecal microbiota is likely to be an important aggravating factor of cryptosporidiosis in calves. This novel finding may contribute to the development of improved control strategies for cryptosporidiosis.

## Methods

### Ethics statement

This study was performed in strict accordance with recommendations in the Guide for the Care and Use of Laboratory Animals of Ministry of Educations, Culture Sports, Science and Technology, Japan. The protocol was approved by the Committee on the Ethics of Animal Experiments of Iwate University (Permit number A201536).

### Faecal sample collection from farm #A for metagenomic analysis

Faecal samples were collected from 20 neonatal Holstein calves at a farm (farm #A) in Iwate Prefecture, Japan, where cryptosporidiosis caused by *C. parvum* had consistently been reported in neonatal calves^39^. To achieve consistent sampling quality, all of the calves were housed and handled in the same way as much as possible. All of the calves were female and were born in identical cattle sheds. Birth weights ranged from 35–45 kg. Premature calves were excluded from the sampling. No vaccination programme was implemented for neonatal calves from farm #A. Within 2 h of birth, calves were fed 2 litres of colostrum, followed by 3 litres of colostrum twice daily for 4 days. An equivalent amount of a milk substitute (Pote-Mow Milk; NOSAN, Yokohama, Japan) was used thereafter. A calf starter (Bio calf Neo, NOSAN) was fed to all calves during this period.

Faecal samples were collected from each calf every two or three days between birth and 15 days of age, resulting in a total of 120 faecal samples from farm #A across six sampling points. Each sample was divided between two plastic tubes. From the first tube, half of each sample was analysed for the presence of *C. parvum*, rotavirus, coronavirus, and *Escherichia coli* K99 antigens using commercial immunochromatographic test (ICT) strips (Bio-X Diagnostics SPRL, Jemelle, Belgium). The remaining half of each faecal sample from the first tube was used to calculate the abundance of oocysts, as described below. The faecal score for each sample was recorded at the same time point, with the severity of diarrhoea scored according to the appearance of faecal material: normal (0), loose (1), muddy (2), and watery (3). The second tube from each sample was preserved at −80 °C for DNA extraction.

### Faecal sample collection from farms in different locations

To examine the correlation between *C. parvum* and the faecal microbiome in the neonatal calves from different origins, 23 faecal samples were collected from calves aged 11–15 days born at farms located in Okinawa, Kagoshima, Iwate and Hokkaido prefectures, Japan (Supplementary Fig. S1). The ages of the calves were very similar to those of calves from farm #A at the 6^th^ sampling point (10–15 days old). The faecal samples were preserved at −20 °C for DNA extraction.

### DNA extraction

A Powersoil DNA Isolation Kit (MoBio Laboratories Inc., Carlsbad, CA, USA) was used to isolate DNA from the faecal samples from farm #A and from the different locations in Iwate and Hokkaido prefectures. A QIAamp DNA Stool Mini Kit (Qiagen, Hilden, Germany) was used for DNA extraction from the samples from Okinawa and Kagoshima prefectures.

### Metagenomic analysis of samples from farm #A

Each library was prepared with a primer set (784 F:5′-AGGATTAGATACCCTGGTA-3′ and 1061 R: 5′-CRRCACGAGCTGACGAC-3′3′) targeting the V5-V6 region of the 16 S rRNA genes of bacterium and an Ion Plus Fragment Library Kit (Life Technologies). Sequencing was performed using a 318 chip and an Ion PGM Sequencing 400 Kit (Life Technologies) on the Ion PGM sequencer (Life Technologies). Raw sequences were demultiplexed and quality-trimmed using the following procedures: (i) trimming of bases with a quality less than Q15 from the 3ʹ end of each read, (ii) removal of reads with an average quality less than Q20, (iii) removal of reads without primer sequences at both ends and (iv) removal of reads with a total length less than 260 bp. These steps were carried out using the FASTX-Toolkit (http://hannonlab.cshl.edu/fastx_toolkit/index.html) and BBtrim (http://bbmap.sourceforge.net/). Subsequently, 10,000 reads per sample were randomly sampled, using the random_sequence_sample.pl (https://www.ualberta.ca/~stothard/software.html) algorithm for taxonomic assignment. These sequences were then clustered into operational taxonomic units (OTU) defined at 97% similarity cutoff using UCLUST version 1.2.22q. Representative sequences for each OTU were classified taxonomically using RDP Classifier version 2.2 with the greengenes database (gg_13_8).

### qPCR analysis

qPCR assays were carried out to determine the abundance of *C. parvum* and *Fusobacterium* sequences in each of the samples. Faecal samples collected at the 6^th^ sampling point from farm #A along with the 23 samples from different origins were used for qPCR analyses.

To determine the abundance of *C. parvum* sequences, a qPCR targeting the 18 S rRNA gene was performed with the following primers: forward, 5′-CTCGACTTTATGGAAGGGTTG-3′; reverse, 5′-CAGAAACTTGAATGATATGTCACATTTAA-3′^40^. Reaction mixtures were prepared to a final volume of 20 µl and contained 0.2 µM of each primer, 0.4 µl of 50 × ROX reference dye, 10 µl of SYBR qPCR Mix (THUNDERBIRD SYBR qPCR Mix, TOYOBO Co., Osaka, Japan) and template DNA. The qPCR was performed using a StepOnePlus Real-Time PCR System (Applied Biosystems, Foster City, CA, USA) with an initial denaturation step of 95 °C for 60 s, followed by 35 cycles of 95 °C for 15 s and 64 °C for 30 s.

To determine the abundance of *Fusobacterium* sequences, a qPCR targeting the 16 S rRNA gene was performed using the following primers: forward, 5′-C(A/T)AACGCGATAAGTAATC-3′; reverse, 5′-TGGTAACATACGA(A/T)AGGG-3′^41^. The composition of the reaction mixture was identical to that described above. The thermal cycler conditions consisted of an initial denaturation step of 95 °C for 60 s followed by 40 cycles of 95 °C for 15 s and 60 °C for 30 s.

The amount of template DNA varied according to the quality of DNA: 1.6 ng for samples from farm #A, 4.0 ng for samples from Okinawa and Kagoshima prefectures and 40 ng for the samples from Iwate and Hokkaido prefectures.

Control plasmids containing the 18 S rRNA and 16 S rRNA gene sequences from *C. parvum* and *Fusobacterium*, respectively, were constructed and used to generate standard curves for the qPCR assays. Briefly, each PCR product from the respective targets was cloned into pUC118 using a Mighty Cloning Reagent Set (Blunt End) (TaKaRa Bio Inc., Otsu, Japan). The resultant plasmids were then linearised by digestion with *Eco*RI.

The *C. parvum* and *Fusobacterium* loads in each sample (copy number/ng DNA) were determined based on a standard calibration curve generated from qPCR assays carried out using a dilution series (10^3^–10^8^ copies/μl) of each of the control plasmids. qPCR assays were repeated at least twice to confirm the reproducibility of the results.

### Calculation of oocyst numbers in faecal samples from farm #A

Oocyst numbers in the *C. parvum-*positive samples identified by ICT strips were determined using the sugar flotation method, as described previously^42^. Briefly, 1 g of faecal sample was resuspended in water and centrifuged at 2,000 × *g* for 10 min. The supernatant was discarded, and the pellet resuspended in sucrose solution (1.2 g/ml) before being centrifuged using the same conditions as above. Following centrifugation, oocysts present in the sample floated to the top of the supernatant and could be counted to give the approximate number of oocysts per gram (OPG). The average number of oocysts in 10 fields at a magnification of 400× was calculated under an optical microscope, with the average number converted into OPG by using the total number of fields under the microscope. The total number of fields for the optical microscope used in this study (BX51; Olympus, Tokyo, Japan) was 1,089.

### Statistical analysis

Statistical analyses were performed using GraphPad Prism version 7.04 (GraphPad Software Inc.).

Wilcoxon test was performed to compare the distribution of bacterial relative abundance in samples from in farm #A. The differences between the 1^st^ and the 6^th^ sampling points were examined respectively in the three groups: *C. parvum*-only-infected, rotavirus-only-infected, and the pathogen negative samples.

Correlation coefficients were calculated to examine the relationship between the number of *C. parvum* oocysts and the relative ratios of *Fusobacterium*, as well as between the DNA copy numbers of *C. parvum* and *Fusobacterium*.

The associations between *C. parvum* and *Fusobacterium*, based on the number of positive and negative samples for each determined by qPCR analysis of the faecal samples from different origins, were examined by Fisher’s exact test.

## Supplementary information

Supplementary material

## Data Availability

All data generated or analysed during this study are included in this published article (and its Supplementary Information Files).
