# Supplementary material for: Specific increase of Fusobacterium in the faecal microbiota of neonatal calves infected with Cryptosporidium parvum
Source: Sci Rep. 2019 Aug 29;9:12517. doi: 10.1038/s41598-019-48969-6 (PMC6715637; doi:10.1038/s41598-019-48969-6)

Specific increase of *Fusobacterium* in the faecal microbiota of neonatal calves infected with *Cryptosporidium parvum*

Madoka Ichikawa-Seki[a], Daisuke Motooka[b], Aiko Kinami[c], Fumi Murakoshi[d,e], Yoko Takahashi[f], Junya Aita[a], Kei Hayashi[a,g], Atsushi Tashibu[a], Shota Nakamura[b], Tetsuya Iida[b], Toshihiro Horii[b], Yoshifumi Nishikawa[d*]

[a]Laboratory of Veterinary Parasitology, Faculty of Agriculture, Iwate University, 3-18-8 Ueda, Morioka 020-8550, Japan; [b]Department of Infection Metagenomics, Genome Information Research Center, Research Institute for Microbial Diseases, Osaka University, Suita 565-0871, Japan; [c]Famille202, 110-16, Ogama-todate, Takizawa, Iwate 020-0762, Japan; [d]National Research Center for Protozoan Diseases, Obihiro University of Agriculture and Veterinary Medicine, Nishi 2-13 Inada-cho, Obihiro 080-8555, Japan; [e]Department of Infectious Diseases, Kyoto Prefectural School of Medicine, 465, Kajiicho, Kawaramachi-hirokoji, Kamigyo-ku, Kyoto, 602-8566, Japan; [f]Tyubu Area Center Veterinary Clinic, Iwate Agricultural Mutual Aid Association, 821 Shimoneko, Hanamaki, Iwate 025-0025, Japan; [g]Laboratory of Veterinary Parasitology, Faculty of Veterinary Medicine, Okayama University of Science, 1-3 Ikoinooka, Imabari 794-8555, Japan.

*Address correspondence to Yoshifumi Nishikawa, nisikawa@obihiro.ac.jp

Supplementary material

Table S1 Age in days of six time collection for the 20 calves in farm #A.

Group	Neonatal calf ID	Age in days															
		0	1	2	3	4	5	6	7	8	9	10	11	12	13	14	15
C. parvum -only infected (*n* = 8)	1		1.1			1.2			1.3			1.4			1.5		1.6
	2		2.1		2.2			2.3		2.4			2.5		2.6		
	6		6.1			6.2		6.3		6.4			6.5		6.6		
	8		8.1		8.2			8.3		8.4		8.5			8.6		
	12		12.1		12.2			12.3			12.4			12.5			12.6
	21		21.1			21.2			21.3			21.4		21.5		21.6	
	22		22.1		22.2		22.3		22.4		22.5		22.6				
	25		25.1		25.2			25.3			25.4		25.5		25.6		
total sample number		0	8	0	5	3	1	5	3	3	3	3	4	2	5	1	2
rotavirus-only infected (*n* = 5)	28		28.1				28.2			28.3		28.4		28.5			28.6
	30		30.1		30.2			30.3		30.4		30.5		30.6			
	31		31.1		31.2			31.3		31.4		31.5		31.6			
	35		35.1			35.2		35.3		35.4			35.5		35.6		
	37		37.1		37.2			37.3		37.4		37.5			37.6		
total sample number		0	5	0	3	1	1	4	0	5	0	4	1	3	2	0	1
pathogen-negative calves (n = 7)	26		26.1		26.2		26.3				26.4			26.5		26.6	
	29		29.1			29.2			29.3		29.4		29.5		29.6		
	32		32.1		32.2		32.3		32.4		32.5		32.6				
	33		33.1		33.2		33.3		33.4		33.5		33.6				
	34	34.1		34.2		34.3		34.4		34.5		34.6					
	36		36.1			36.2			36.3		36.4		36.5			36.6	
	38		38.1			38.2		38.3			38.4		38.5		38.6		
total sample number		1	6	1	3	4	3	2	4	1	6	1	5	1	2	2	0

The number of each colloection was shown after a dot.

Table S2 Distribution of bacterial relative abundance (%) among the three groups from farm #A.

	genus	*C. parvum* (*n* = 8)	Rotavirus (*n* = 5)	Negative (*n* = 7)
1	Bacteria;__Fusobacteria;__Fusobacteria;__Fusobacteriales;__Fusobacteriaceae;__Fusobacterium	14.1	0.7	2.0
2	Bacteria;__Proteobacteria;__Gammaproteobacteria;__Enterobacteriales;__Enterobacteriaceae;__Escherichia-Shigella	12.8	11.9	17.0
3	Bacteria;__Bacteroidetes;__Bacteroidia;__Bacteroidales;__Bacteroidaceae;__Bacteroides	10.8	9.3	15.3
4	Bacteria;__Bacteroidetes;__Bacteroidia;__Bacteroidales;__Prevotellaceae;__Prevotella	8.1	12.1	9.2
5	Bacteria;__Firmicutes;__Bacilli;__Lactobacillales;__Lactobacillaceae;__Lactobacillus	7.9	10.3	9.2
6	Bacteria;__Bacteroidetes;__Bacteroidia;__Bacteroidales;__ratAN060301C;__g	5.6	7.7	9.2
7	Bacteria;__Firmicutes;__Clostridia;__Clostridiales;__Ruminococcaceae;__Faecalibacterium	4.5	6.8	7.3
8	Bacteria;__Bacteroidetes;__Bacteroidia;__Bacteroidales;__Prevotellaceae;Other	3.8	2.1	1.0
9	Bacteria;__Firmicutes;__Clostridia;__Clostridiales;__Lachnospiraceae;Other	3.7	4.1	4.3
10	Bacteria;__Firmicutes;__Clostridia;__Clostridiales;__Ruminococcaceae;__Incertae_Sedis	3.4	1.7	2.6
11	Bacteria;Other;Other;Other;Other;Other	3.2	1.6	1.0
12	Bacteria;__Firmicutes;__Clostridia;__Clostridiales;__Clostridiaceae;__Clostridium	2.8	0.7	2.5
13	Bacteria;__Proteobacteria;__Betaproteobacteria;__Burkholderiales;__Alcaligenaceae;__Sutterella	2.4	1.8	2.6
14	Bacteria;__Proteobacteria;__Gammaproteobacteria;__Enterobacteriales;__Enterobacteriaceae;Other	2.1	5.5	2.2
15	Bacteria;__Proteobacteria;__Gammaproteobacteria;__Pasteurellales;__Pasteurellaceae;__Gallibacterium	2.0	1.6	1.3
16	Bacteria;__Firmicutes;__Bacilli;__Lactobacillales;__Enterococcaceae;__Enterococcus	1.3	0.8	1.6
17	Bacteria;__Actinobacteria;__Coriobacteriia;__Coriobacteriales;__Coriobacteriaceae;__Collinsella	1.2	1.1	0.9
18	Bacteria;__Proteobacteria;__Gammaproteobacteria;__Pasteurellales;__Pasteurellaceae;Other	1.1	1.1	0.9
19	Bacteria;__Firmicutes;__Clostridia;__Clostridiales;__Lachnospiraceae;__Blautia	1.0	2.8	1.6
20	Bacteria;__Bacteroidetes;__Bacteroidia;__Bacteroidales;Other;Other	0.9	0.6	0.4
21	Bacteria;__Bacteroidetes;__Bacteroidia;__Bacteroidales;__Porphyromonadaceae;__Parabacteroides	0.7	0.8	0.4
22	Bacteria;__Firmicutes;__Bacilli;__Lactobacillales;Other;Other	0.6	0.6	0.3
23	Bacteria;__Proteobacteria;__Epsilonproteobacteria;__Campylobacterales;__Campylobacteraceae;__Campylobacter	0.5	0.1	0.0
24	Bacteria;__Proteobacteria;__Gammaproteobacteria;__Enterobacteriales;__Enterobacteriaceae;__Proteus	0.5	0.0	0.0
25	Bacteria;__Actinobacteria;__Actinobacteria;__Bifidobacteriales;__Bifidobacteriaceae;Other	0.5	2.7	0.9
26	Bacteria;__Firmicutes;__Clostridia;__Clostridiales;__Lachnospiraceae;__Incertae_Sedis	0.4	0.9	0.4
27	Bacteria;__Proteobacteria;__Gammaproteobacteria;__Aeromonadales;__Succinivibrionaceae;__Succinivibrio	0.4	2.1	1.2
28	Bacteria;__Firmicutes;__Clostridia;__Clostridiales;__Lachnospiraceae;__Dorea	0.3	1.4	0.7
29	Bacteria;__Firmicutes;__Clostridia;__Clostridiales;__Peptostreptococcaceae;__Incertae_Sedis	0.3	0.6	0.5
30	Bacteria;__Firmicutes;__Bacilli;__Lactobacillales;__Streptococcaceae;__Streptococcus	0.3	0.4	0.3
31	Bacteria;__Firmicutes;Other;Other;Other;Other	0.2	0.2	0.2
32	Bacteria;__Firmicutes;__Clostridia;__Clostridiales;__Veillonellaceae;__Allisonella	0.2	0.2	0.2
33	Bacteria;__Firmicutes;__Clostridia;__Clostridiales;__Veillonellaceae;__Anaerovibrio	0.2	0.1	0.0
34	Bacteria;__Actinobacteria;__Actinobacteria;__Bifidobacteriales;__Bifidobacteriaceae;__Gardnerella	0.2	0.3	0.4
35	Bacteria;__Firmicutes;__Clostridia;__Clostridiales;__Ruminococcaceae;__Subdoligranulum	0.2	0.6	0.1
36	Bacteria;__Bacteroidetes;__Bacteroidia;__Bacteroidales;__Rikenellaceae;__RC9_gut_group	0.1	0.2	0.1
37	Bacteria;__Firmicutes;__Clostridia;__Clostridiales;__Veillonellaceae;Other	0.1	0.2	0.0
38	Bacteria;__Proteobacteria;__Betaproteobacteria;__Burkholderiales;__Alcaligenaceae;Other	0.1	0.0	0.0
39	Bacteria;__Firmicutes;__Clostridia;__Clostridiales;__Peptostreptococcaceae;__Peptostreptococcus	0.1	0.0	0.0
40	Bacteria;__Firmicutes;__Clostridia;__Clostridiales;__Veillonellaceae;__Megamonas	0.1	0.0	0.0
41	Bacteria;__Actinobacteria;__Actinobacteria;__Bifidobacteriales;__Bifidobacteriaceae;__Bifidobacterium	0.1	0.0	0.0
42	Bacteria;__Proteobacteria;__Deltaproteobacteria;__Desulfovibrionales;__Desulfovibrionaceae;__Desulfovibrio	0.1	0.0	0.0
43	Bacteria;__Firmicutes;__Clostridia;__Clostridiales;__Lachnospiraceae;__Moryella	0.1	0.0	0.1
44	Bacteria;__Firmicutes;__Clostridia;__Clostridiales;__Ruminococcaceae;Other	0.1	0.1	0.1
45	Bacteria;__Firmicutes;__Clostridia;__Clostridiales;__Lachnospiraceae;__Marvinbryantia	0.1	0.1	0.2
46	Bacteria;__Proteobacteria;__Gammaproteobacteria;__Aeromonadales;__Aeromonadaceae;__Aeromonas	0.1	0.0	0.0
47	Bacteria;__Bacteroidetes;__Bacteroidia;__Bacteroidales;__Prevotellaceae;__g	0.1	0.0	0.0
48	Bacteria;__Bacteroidetes;__Bacteroidia;__Bacteroidales;__Porphyromonadaceae;__Odoribacter	0.1	0.0	0.0
49	Bacteria;__Proteobacteria;__Gammaproteobacteria;__Pasteurellales;__Pasteurellaceae;__Mannheimia	0.0	0.0	0.0
50	Bacteria;__Proteobacteria;__Gammaproteobacteria;__Pasteurellales;__Pasteurellaceae;__Pasteurella	0.0	0.0	0.0
51	Bacteria;__Bacteroidetes;__Bacteroidia;__Bacteroidales;__S24-7;__g	0.0	0.1	0.0
52	Bacteria;__Firmicutes;__Erysipelotrichi;__Erysipelotrichales;__Erysipelotrichaceae;__Turicibacter	0.0	0.0	0.0
53	Bacteria;__Actinobacteria;__Coriobacteriia;__Coriobacteriales;__Coriobacteriaceae;__Atopobium	0.0	0.1	0.0
54	Bacteria;__Firmicutes;__Clostridia;__Clostridiales;__Veillonellaceae;__Veillonella	0.0	0.0	0.0
55	Bacteria;__Proteobacteria;__Betaproteobacteria;__Burkholderiales;__Alcaligenaceae;__Parasutterella	0.0	0.1	0.0
56	Bacteria;__Firmicutes;__Clostridia;__Clostridiales;__Clostridiaceae;Other	0.0	0.0	0.0
57	Bacteria;__Firmicutes;__Clostridia;__Clostridiales;__Lachnospiraceae;__Howardella	0.0	0.0	0.0
58	Bacteria;__Bacteroidetes;__Bacteroidia;__Bacteroidales;__Rikenellaceae;__Alistipes	0.0	0.0	0.0
59	Bacteria;__Proteobacteria;__Gammaproteobacteria;__Enterobacteriales;__Enterobacteriaceae;__Klebsiella	0.0	0.0	0.1
60	Bacteria;__Bacteroidetes;__Bacteroidia;__Bacteroidales;__Porphyromonadaceae;__Butyricimonas	0.0	0.0	0.0
61	Bacteria;__Fusobacteria;__Fusobacteria;__Fusobacteriales;__Fusobacteriaceae;Other	0.0	0.0	0.0
62	Bacteria;__Firmicutes;__Clostridia;__Clostridiales;__Family_XIII_Incertae_Sedis;__Mogibacterium	0.0	0.0	0.0
63	Bacteria;__Firmicutes;__Clostridia;__Clostridiales;__Veillonellaceae;__Mitsuokella	0.0	0.0	0.0
64	Bacteria;__Firmicutes;__Clostridia;__Clostridiales;__Ruminococcaceae;__Anaerotruncus	0.0	0.0	0.0
65	Bacteria;__Actinobacteria;__Actinobacteria;__Corynebacteriales;Other;Other	0.0	0.0	0.0
66	Bacteria;__Firmicutes;__Clostridia;__Clostridiales;__Peptostreptococcaceae;Other	0.0	0.0	0.0
67	Bacteria;__Firmicutes;__Clostridia;__Clostridiales;__Clostridiaceae;__Sarcina	0.0	0.2	0.1
68	Bacteria;__Firmicutes;__Clostridia;__Clostridiales;__Veillonellaceae;__Phascolarctobacterium	0.0	0.1	0.0
69	Bacteria;__Firmicutes;__Bacilli;Other;Other;Other	0.0	0.0	0.0
70	Bacteria;__Firmicutes;__Clostridia;__Clostridiales;__Lachnospiraceae;__Syntrophococcus	0.0	0.0	0.0
71	Bacteria;__Actinobacteria;__Actinobacteria;__Actinomycetales;__Actinomycetaceae;__Actinomyces	0.0	0.0	0.0
72	Bacteria;__Fusobacteria;__Fusobacteria;__Fusobacteriales;Other;Other	0.0	0.0	0.0
73	Bacteria;__Proteobacteria;__Gammaproteobacteria;__Aeromonadales;__Succinivibrionaceae;Other	0.0	0.0	0.0
74	Bacteria;__Firmicutes;__Bacilli;__Lactobacillales;__Lactobacillaceae;Other	0.0	0.0	0.0
75	Bacteria;__Proteobacteria;__Betaproteobacteria;__Burkholderiales;__Comamonadaceae;Other	0.0	0.2	0.1
76	Bacteria;__Bacteroidetes;__Bacteroidia;__Bacteroidales;__Porphyromonadaceae;Other	0.0	0.0	0.0
77	Bacteria;__Actinobacteria;__Coriobacteriia;__Coriobacteriales;__Coriobacteriaceae;Other	0.0	0.0	0.0
78	Bacteria;__Firmicutes;__Clostridia;__Clostridiales;Other;Other	0.0	0.0	0.0
79	Bacteria;__Proteobacteria;__Betaproteobacteria;__Burkholderiales;Other;Other	0.0	0.0	0.0
80	Bacteria;__Proteobacteria;__Gammaproteobacteria;Other;Other;Other	0.0	0.0	0.0
81	Bacteria;__Firmicutes;__Clostridia;__Clostridiales;__Lachnospiraceae;__Pseudobutyrivibrio	0.0	0.0	0.0
82	Bacteria;__Firmicutes;__Clostridia;__Clostridiales;__Ruminococcaceae;__g	0.0	0.1	0.0
83	Bacteria;__Firmicutes;__Clostridia;__Clostridiales;__Family_XIII_Incertae_Sedis;__Incertae_Sedis	0.0	0.0	0.0
84	Bacteria;__Firmicutes;__Clostridia;__Clostridiales;__Lachnospiraceae;__Butyrivibrio	0.0	0.0	0.0
85	Bacteria;__Actinobacteria;__Actinobacteria;__Corynebacteriales;__Corynebacteriaceae;__Corynebacterium	0.0	0.0	0.0
86	Bacteria;__Firmicutes;__Clostridia;__Clostridiales;__Ruminococcaceae;__Papillibacter	0.0	0.0	0.0
87	Bacteria;__Firmicutes;__Erysipelotrichi;__Erysipelotrichales;__Erysipelotrichaceae;__Incertae_Sedis	0.0	0.0	0.0

88	Bacteria;__Proteobacteria;__Gammaproteobacteria;__Aeromonadales;__Succinivibrionaceae;__g	0.0	0.0	0.0
89	Bacteria;__Firmicutes;__Clostridia;__Clostridiales;__Veillonellaceae;__Acidaminococcus	0.0	0.0	0.0
90	Bacteria;__Bacteroidetes;__Bacteroidia;__Bacteroidales;__p-2534-18B5_gut_group;__g	0.0	0.0	0.0
91	Bacteria;__Bacteroidetes;__Bacteroidia;__Bacteroidales;__Rikenellaceae;Other	0.0	0.0	0.0
92	Bacteria;__Firmicutes;__Clostridia;__Clostridiales;__Family_XI_Incertae_Sedis;__Parvimonas	0.0	0.0	0.0
93	Bacteria;__Firmicutes;__Clostridia;__Clostridiales;__Peptococcaceae;__Peptococcus	0.0	0.0	0.0
94	Bacteria;__Firmicutes;__Clostridia;__Clostridiales;__Family_XI_Incertae_Sedis;__Anaerococcus	0.0	0.0	0.0
95	Bacteria;__Proteobacteria;__Gammaproteobacteria;__Pseudomonadales;__Moraxellaceae;__Acinetobacter	0.0	0.1	0.0
96	Bacteria;__Firmicutes;__Clostridia;__Clostridiales;__Eubacteriaceae;__Eubacterium	0.0	0.0	0.0
97	Bacteria;__Proteobacteria;__Deltaproteobacteria;__Desulfovibrionales;__Desulfovibrionaceae;__Bilophila	0.0	0.0	0.0
98	Bacteria;__Proteobacteria;__Gammaproteobacteria;__Pseudomonadales;__Pseudomonadaceae;Other	0.0	0.0	0.0
99	Bacteria;__Firmicutes;__Clostridia;__Clostridiales;__Veillonellaceae;__Dialister	0.0	0.0	0.0
100	Bacteria;__Actinobacteria;__Actinobacteria;__Actinomycetales;__Actinomycetaceae;__Trueperella	0.0	0.0	0.0
101	Bacteria;__Firmicutes;__Clostridia;__Clostridiales;__Family_XIII_Incertae_Sedis;Other	0.0	0.0	0.0
102	Bacteria;__Lentisphaerae;__Lentisphaeria;__Victivallales;__Victivallaceae;__Victivallis	0.0	0.0	0.0
103	Bacteria;__Proteobacteria;__Deltaproteobacteria;__Desulfovibrionales;__Desulfovibrionaceae;Other	0.0	0.0	0.0
104	Bacteria;__Fusobacteria;__Fusobacteria;__Fusobacteriales;__CFT112H7;__g	0.0	0.0	0.0
105	Bacteria;__Actinobacteria;__Coriobacteriia;__Coriobacteriales;__Coriobacteriaceae;__Eggerthella	0.0	0.0	0.0
106	Bacteria;__Bacteroidetes;__Bacteroidia;__Bacteroidales;__Porphyromonadaceae;__Porphyromonas	0.0	0.0	0.0
107	Bacteria;__Firmicutes;__Bacilli;__Bacillales;__Planococcaceae;__Lysinibacillus	0.0	0.0	0.1
108	Bacteria;__Proteobacteria;__Gammaproteobacteria;__Pseudomonadales;__Moraxellaceae;__Psychrobacter	0.0	0.0	0.0
109	Bacteria;__Proteobacteria;Other;Other;Other;Other	0.0	0.0	0.0
110	Bacteria;__Bacteroidetes;Other;Other;Other;Other	0.0	0.0	0.0
111	Bacteria;__Firmicutes;__Bacilli;__Lactobacillales;__Streptococcaceae;__Lactococcus	0.0	0.0	0.0
112	Bacteria;__Firmicutes;__Clostridia;__Clostridiales;__Family_XI_Incertae_Sedis;__Helcococcus	0.0	0.0	0.0
113	Bacteria;__Firmicutes;__Clostridia;__Clostridiales;__Veillonellaceae;__Megasphaera	0.0	0.1	0.0
114	Bacteria;__Fusobacteria;__Fusobacteria;__Fusobacteriales;__boneC3G7;__g	0.0	0.0	0.0
115	Bacteria;__Proteobacteria;__Alphaproteobacteria;Other;Other;Other	0.0	0.0	0.0
116	Bacteria;__Actinobacteria;__Actinobacteria;__Actinomycetales;__Actinomycetaceae;Other	0.0	0.0	0.0
117	Bacteria;__Proteobacteria;__Epsilonproteobacteria;__Campylobacterales;__Helicobacteraceae;__Helicobacter	0.0	0.0	0.0
118	Bacteria;__Firmicutes;__Clostridia;__Clostridiales;__Veillonellaceae;__Negativicoccus	0.0	0.0	0.0
119	Bacteria;__Firmicutes;__Erysipelotrichi;__Erysipelotrichales;__Erysipelotrichaceae;Other	0.0	0.0	0.0
120	Bacteria;__Proteobacteria;__Betaproteobacteria;Other;Other;Other	0.0	0.0	0.0
121	Bacteria;__Actinobacteria;__Actinobacteria;__Micrococcales;__Micrococcaceae;__Nesterenkonia	0.0	0.0	0.0
122	Bacteria;__Bacteroidetes;__Bacteroidia;__Bacteroidales;__Porphyromonadaceae;__Barnesiella	0.0	0.0	0.0
123	Bacteria;__Bacteroidetes;__Flavobacteria;__Flavobacteriales;__Flavobacteriaceae;Other	0.0	0.0	0.0
124	Bacteria;__Firmicutes;__Clostridia;__Clostridiales;__Family_XI_Incertae_Sedis;__Finegoldia	0.0	0.0	0.0
125	Bacteria;__Actinobacteria;__Actinobacteria;__Corynebacteriales;__Dietziaceae;__Dietzia	0.0	0.0	0.0
126	Bacteria;__Bacteroidetes;__Flavobacteria;__Flavobacteriales;__Flavobacteriaceae;__Weeksella	0.0	0.0	0.0
127	Bacteria;__Proteobacteria;__Gammaproteobacteria;__Vibrionales;__Vibrionaceae;Other	0.0	0.0	0.0
128	Bacteria;__Firmicutes;__Erysipelotrichi;__Erysipelotrichales;__Erysipelotrichaceae;__Sharpea	0.0	0.0	0.0
129	Bacteria;__Actinobacteria;__Actinobacteria;Other;Other;Other	0.0	0.0	0.0
130	Bacteria;__Actinobacteria;__Actinobacteria;__Corynebacteriales;__Nocardiaceae;__Rhodococcus	0.0	0.0	0.0
131	Bacteria;__Bacteroidetes;__Flavobacteria;__Flavobacteriales;Other;Other	0.0	0.0	0.0
132	Bacteria;__Proteobacteria;__Alphaproteobacteria;__Rhodospirillales;__Rhodospirillaceae;__Thalassospira	0.0	0.0	0.0
133	Bacteria;__Proteobacteria;__Gammaproteobacteria;__Pseudomonadales;__Pseudomonadaceae;__Pseudomonas	0.0	0.0	0.0
134	Bacteria;__Proteobacteria;__Betaproteobacteria;__Burkholderiales;__Alcaligenaceae;__Oligella	0.0	0.1	0.0
135	Bacteria;__Actinobacteria;Other;Other;Other;Other	0.0	0.0	0.0
136	Bacteria;__Actinobacteria;__Actinobacteria;__Propionibacteriales;__Propionibacteriaceae;__Propionibacterium	0.0	0.1	0.1
137	Bacteria;__Bacteroidetes;__Bacteroidia;__Bacteroidales;__Porphyromonadaceae;__Dysgonomonas	0.0	0.0	0.0
138	Bacteria;__Firmicutes;__Clostridia;__Clostridiales;__Eubacteriaceae;__Pseudoramibacter	0.0	0.0	0.0
139	Bacteria;__Firmicutes;__Clostridia;__Clostridiales;__Family_XI_Incertae_Sedis;__Peptoniphilus	0.0	0.0	0.0
140	Bacteria;__Firmicutes;__Clostridia;__Clostridiales;__Ruminococcaceae;__Anaerofilum	0.0	0.0	0.0
141	Bacteria;__Firmicutes;__Clostridia;__Clostridiales;__Ruminococcaceae;__Flavonifractor	0.0	0.0	0.0
142	Bacteria;__Proteobacteria;__Alphaproteobacteria;__Caulobacterales;__Caulobacteraceae;__Brevundimonas	0.0	0.0	0.0
143	Bacteria;__Proteobacteria;__Alphaproteobacteria;__Rhizobiales;__Brucellaceae;__Ochrobactrum	0.0	0.0	0.0
144	Bacteria;__Proteobacteria;__Alphaproteobacteria;__Sphingomonadales;__Sphingomonadaceae;Other	0.0	0.4	0.2
145	Bacteria;__Proteobacteria;__Betaproteobacteria;__Burkholderiales;__Oxalobacteraceae;__Undibacterium	0.0	0.0	0.0
146	Bacteria;__Proteobacteria;__Betaproteobacteria;__Neisseriales;__Neisseriaceae;Other	0.0	0.0	0.0
147	Bacteria;__Proteobacteria;__Gammaproteobacteria;__Pseudomonadales;__Moraxellaceae;__Alkanindiges	0.0	0.0	0.0
148	Bacteria;__Proteobacteria;__Gammaproteobacteria;__Xanthomonadales;__Xanthomonadaceae;__Stenotrophomonas	0.0	0.1	0.0
149	Bacteria;__Actinobacteria;__Actinobacteria;__Actinomycetales;__Actinomycetaceae;__Mobiluncus	0.0	0.0	0.0
150	Bacteria;__Actinobacteria;__Actinobacteria;__Bifidobacteriales;__Bifidobacteriaceae;__Alloscardovia	0.0	0.0	0.0
151	Bacteria;__Actinobacteria;__Actinobacteria;__Micrococcales;Other;Other	0.0	0.0	0.0
152	Bacteria;__Actinobacteria;__Actinobacteria;__Micrococcales;__Microbacteriaceae;__Amnibacterium	0.0	0.0	0.0
153	Bacteria;__Actinobacteria;__Actinobacteria;__Micrococcales;__Microbacteriaceae;__Curtobacterium	0.0	0.0	0.0
154	Bacteria;__Actinobacteria;__Actinobacteria;__Micrococcales;__Microbacteriaceae;__Microbacterium	0.0	0.0	0.0
155	Bacteria;__Actinobacteria;__Actinobacteria;__Micrococcales;__Micrococcaceae;Other	0.0	0.0	0.0
156	Bacteria;__Actinobacteria;__Actinobacteria;__Micrococcales;__Micrococcaceae;__Micrococcus	0.0	0.0	0.0
157	Bacteria;__Actinobacteria;__Actinobacteria;__Micrococcales;__Micrococcaceae;__Rothia	0.0	0.0	0.0
158	Bacteria;__Actinobacteria;__Actinobacteria;__Streptomycetales;__Streptomycetaceae;__Streptomyces	0.0	0.0	0.0
159	Bacteria;__Actinobacteria;__Coriobacteriia;__Coriobacteriales;__Coriobacteriaceae;__g	0.0	0.0	0.0
160	Bacteria;__Bacteroidetes;__Flavobacteria;__Flavobacteriales;__Flavobacteriaceae;__Bergeyella	0.0	0.0	0.0
161	Bacteria;__Bacteroidetes;__Flavobacteria;__Flavobacteriales;__Flavobacteriaceae;__Capnocytophaga	0.0	0.0	0.0
162	Bacteria;__Bacteroidetes;__Flavobacteria;__Flavobacteriales;__Flavobacteriaceae;__Myroides	0.0	0.0	0.0
163	Bacteria;__Bacteroidetes;__Sphingobacteriia;__Sphingobacteriales;__Chitinophagaceae;__g	0.0	0.0	0.0
164	Bacteria;__Bacteroidetes;__Sphingobacteriia;__Sphingobacteriales;__Sphingobacteriaceae;__Olivibacter	0.0	0.0	0.0
165	Bacteria;__Bacteroidetes;__Sphingobacteriia;__Sphingobacteriales;__Sphingobacteriaceae;__Sphingobacterium	0.0	0.0	0.0
166	Bacteria;__Firmicutes;__Bacilli;__Bacillales;__Planococcaceae;Other	0.0	0.0	0.0
167	Bacteria;__Firmicutes;__Bacilli;__Bacillales;__Planococcaceae;__Sporosarcina	0.0	0.0	0.0
168	Bacteria;__Firmicutes;__Bacilli;__Bacillales;__Staphylococcaceae;__Jeotgalicoccus	0.0	0.0	0.0
169	Bacteria;__Firmicutes;__Bacilli;__Lactobacillales;__Aerococcaceae;__Eremococcus	0.0	0.0	0.0
170	Bacteria;__Firmicutes;__Bacilli;__Lactobacillales;__Carnobacteriaceae;__Atopostipes	0.0	0.0	0.0
171	Bacteria;__Firmicutes;__Bacilli;__Lactobacillales;__MOB164;__g	0.0	0.0	0.0
172	Bacteria;__Firmicutes;__Clostridia;__Clostridiales;__Christensenellaceae;__g	0.0	0.0	0.0
173	Bacteria;__Firmicutes;__Clostridia;__Clostridiales;__Family_XI_Incertae_Sedis;Other	0.0	0.0	0.0
174	Bacteria;__Firmicutes;__Clostridia;__Clostridiales;__Lachnospiraceae;__Anaerostipes	0.0	0.0	0.1
175	Bacteria;__Firmicutes;__Clostridia;__Clostridiales;__Lachnospiraceae;__Cellulosilyticum	0.0	0.0	0.0
176	Bacteria;__Firmicutes;__Clostridia;__Clostridiales;__Lachnospiraceae;__Lachnospira	0.0	0.0	0.0

177	Bacteria;__Firmicutes;__Clostridia;__Clostridiales;__Ruminococcaceae;__Fastidiosipila	0.0	0.0	0.0
178	Bacteria;__Fusobacteria;__Fusobacteria;__Fusobacteriales;__Fusobacteriaceae;__Cetobacterium	0.0	0.0	0.0
179	Bacteria;__Proteobacteria;__Alphaproteobacteria;__Caulobacterales;__Caulobacteraceae;__Asticcacaulis	0.0	0.0	0.0
180	Bacteria;__Proteobacteria;__Alphaproteobacteria;__Rhizobiales;Other;Other	0.0	0.0	0.0
181	Bacteria;__Proteobacteria;__Alphaproteobacteria;__Rhizobiales;__Phyllobacteriaceae;Other	0.0	0.1	0.0
182	Bacteria;__Proteobacteria;__Alphaproteobacteria;__Rhizobiales;__Rhizobiaceae;__Rhizobium	0.0	0.3	0.1
183	Bacteria;__Proteobacteria;__Alphaproteobacteria;__Rickettsiales;__mitochondria;__g	0.0	0.0	0.0
184	Bacteria;__Proteobacteria;__Alphaproteobacteria;__Sphingomonadales;__Sphingomonadaceae;__Novosphingobium	0.0	0.0	0.0
185	Bacteria;__Proteobacteria;__Alphaproteobacteria;__Sphingomonadales;__Sphingomonadaceae;__Sphingomonas	0.0	0.1	0.1
186	Bacteria;__Proteobacteria;__Betaproteobacteria;__Burkholderiales;__Burkholderiaceae;__Ralstonia	0.0	0.0	0.0
187	Bacteria;__Proteobacteria;__Epsilonproteobacteria;__Campylobacterales;__Campylobacteraceae;Other	0.0	0.0	0.0
188	Bacteria;__Synergistetes;__Synergistia;__Synergistales;__Synergistaceae;__Pyramidobacter	0.0	0.0	0.0
189	Eukaryota;Other;Other;Other;Other;Other	0.0	0.0	0.0
190	Bacteria;__Actinobacteria;__Actinobacteria;__Corynebacteriales;__Mycobacteriaceae;__Mycobacterium	0.0	0.0	0.0
191	Bacteria;__Actinobacteria;__Actinobacteria;__Micrococcales;__Brevibacteriaceae;__Brevibacterium	0.0	0.0	0.0
192	Bacteria;__Actinobacteria;__Actinobacteria;__Micrococcales;__Dermacoccaceae;__Dermacoccus	0.0	0.0	0.0
193	Bacteria;__Actinobacteria;__Actinobacteria;__Micrococcales;__Intrasporangiaceae;Other	0.0	0.0	0.0
194	Bacteria;__Actinobacteria;__Actinobacteria;__Micrococcales;__Microbacteriaceae;__Leucobacter	0.0	0.0	0.0
195	Bacteria;__Actinobacteria;__Actinobacteria;__Micromonosporales;__Micromonosporaceae;Other	0.0	0.0	0.0
196	Bacteria;__Actinobacteria;__Actinobacteria;__Pseudonocardiales;__Pseudonocardiaceae;Other	0.0	0.0	0.0
197	Bacteria;__Actinobacteria;__Actinobacteria;__Pseudonocardiales;__Pseudonocardiaceae;__Saccharopolyspora	0.0	0.0	0.0
198	Bacteria;__Actinobacteria;__Actinobacteria;__Streptosporangiales;__Nocardiopsaceae;__Nocardiopsis	0.0	0.0	0.0
199	Bacteria;__Actinobacteria;__Coriobacteriia;__Coriobacteriales;__Coriobacteriaceae;__Paraeggerthella	0.0	0.0	0.0
200	Bacteria;__Actinobacteria;__Coriobacteriia;__Coriobacteriales;__Coriobacteriaceae;__Slackia	0.0	0.0	0.0
201	Bacteria;__Bacteroidetes;__Bacteroidia;__Bacteroidales;__Prevotellaceae;__Paraprevotella	0.0	0.0	0.0
202	Bacteria;__Bacteroidetes;__Bacteroidia;__Bacteroidales;__Prevotellaceae;__Xylanibacter	0.0	0.0	0.0
203	Bacteria;__Bacteroidetes;__Bacteroidia;__Bacteroidales;__RF16;__g	0.0	0.0	0.0
204	Bacteria;__Bacteroidetes;__Bacteroidia;__Bacteroidales;__uncultured;__g	0.0	0.0	0.0
205	Bacteria;__Bacteroidetes;__Cytophagia;__Cytophagales;__Cytophagaceae;__Dyadobacter	0.0	0.0	0.0
206	Bacteria;__Bacteroidetes;__Cytophagia;__Cytophagales;__Cytophagaceae;__Hymenobacter	0.0	0.0	0.0
207	Bacteria;__Bacteroidetes;__Flavobacteria;__Flavobacteriales;__Flavobacteriaceae;__Chryseobacterium	0.0	0.0	0.0
208	Bacteria;__Bacteroidetes;__Flavobacteria;__Flavobacteriales;__Flavobacteriaceae;__Elizabethkingia	0.0	0.0	0.0
209	Bacteria;__Bacteroidetes;__Sphingobacteriia;__Sphingobacteriales;Other;Other	0.0	0.0	0.0
210	Bacteria;__Bacteroidetes;__Sphingobacteriia;__Sphingobacteriales;__Chitinophagaceae;__Chitinophaga	0.0	0.1	0.0
211	Bacteria;__Bacteroidetes;__Sphingobacteriia;__Sphingobacteriales;__Chitinophagaceae;__Flavisolibacter	0.0	0.0	0.0
212	Bacteria;__Bacteroidetes;__Sphingobacteriia;__Sphingobacteriales;__Sphingobacteriaceae;Other	0.0	0.0	0.0
213	Bacteria;__Chlamydiae;__Chlamydiae;__Chlamydiales;__Chlamydiaceae;Other	0.0	0.0	0.0
214	Bacteria;__Elusimicrobia;__Elusimicrobia;Other;Other;Other	0.0	0.0	0.0
215	Bacteria;__Firmicutes;__Bacilli;__Bacillales;Other;Other	0.0	0.0	0.0
216	Bacteria;__Firmicutes;__Bacilli;__Bacillales;__Alicyclobacillaceae;__Tumebacillus	0.0	0.1	0.0
217	Bacteria;__Firmicutes;__Bacilli;__Bacillales;__Bacillaceae;Other	0.0	0.1	0.0
218	Bacteria;__Firmicutes;__Bacilli;__Bacillales;__Bacillaceae;__Anaerobacillus	0.0	0.1	0.0
219	Bacteria;__Firmicutes;__Bacilli;__Bacillales;__Bacillaceae;__Geobacillus	0.0	0.0	0.0
220	Bacteria;__Firmicutes;__Bacilli;__Bacillales;__Paenibacillaceae;__Brevibacillus	0.0	0.0	0.0
221	Bacteria;__Firmicutes;__Bacilli;__Bacillales;__Planococcaceae;__Planococcus	0.0	0.0	0.0
222	Bacteria;__Firmicutes;__Bacilli;__Lactobacillales;__Aerococcaceae;__Globicatella	0.0	0.0	0.0
223	Bacteria;__Firmicutes;__Bacilli;__Lactobacillales;__Aerococcaceae;__Ignavigranum	0.0	0.1	0.0
224	Bacteria;__Firmicutes;__Bacilli;__Lactobacillales;__Leuconostocaceae;__Leuconostoc	0.0	0.0	0.0
225	Bacteria;__Firmicutes;__Clostridia;Other;Other;Other	0.0	0.0	0.0
226	Bacteria;__Firmicutes;__Clostridia;__Clostridiales;__Christensenellaceae;__Christensenella	0.0	0.0	0.0
227	Bacteria;__Firmicutes;__Clostridia;__Clostridiales;__Eubacteriaceae;__Anaerofustis	0.0	0.0	0.0
228	Bacteria;__Firmicutes;__Clostridia;__Clostridiales;__Family_XIII_Incertae_Sedis;__g	0.0	0.0	0.0
229	Bacteria;__Firmicutes;__Clostridia;__Clostridiales;__Lachnospiraceae;__Acetitomaculum	0.0	0.0	0.0
230	Bacteria;__Firmicutes;__Clostridia;__Clostridiales;__Lachnospiraceae;__Roseburia	0.0	0.0	0.0
231	Bacteria;__Firmicutes;__Clostridia;__Clostridiales;__Lachnospiraceae;__g	0.0	0.0	0.0
232	Bacteria;__Firmicutes;__Clostridia;__Clostridiales;__Peptococcaceae;Other	0.0	0.0	0.0
233	Bacteria;__Firmicutes;__Clostridia;__Clostridiales;__Ruminococcaceae;__Oscillospira	0.0	0.0	0.0
234	Bacteria;__Firmicutes;__Clostridia;__Clostridiales;__Veillonellaceae;__Succiniclasticum	0.0	0.0	0.0
235	Bacteria;__Firmicutes;__Clostridia;__Clostridiales;__uncultured;__g	0.0	0.0	0.0
236	Bacteria;__Firmicutes;__Erysipelotrichi;__Erysipelotrichales;__Erysipelotrichaceae;__Solobacterium	0.0	0.0	0.0
237	Bacteria;__Lentisphaerae;__Lentisphaeria;__RFP12_gut_group;__f;__g	0.0	0.0	0.0
238	Bacteria;__Proteobacteria;__Alphaproteobacteria;__Rhizobiales;__Methylobacteriaceae;__Methylobacterium	0.0	0.1	0.0
239	Bacteria;__Proteobacteria;__Alphaproteobacteria;__Rhizobiales;__Rhizobiaceae;Other	0.0	0.0	0.0
240	Bacteria;__Proteobacteria;__Alphaproteobacteria;__Rhodobacterales;__Rhodobacteraceae;Other	0.0	0.0	0.0
241	Bacteria;__Proteobacteria;__Alphaproteobacteria;__Rhodobacterales;__Rhodobacteraceae;__Paracoccus	0.0	0.0	0.0
242	Bacteria;__Proteobacteria;__Alphaproteobacteria;__Sphingomonadales;Other;Other	0.0	0.0	0.0
243	Bacteria;__Proteobacteria;__Alphaproteobacteria;__Sphingomonadales;__Sphingomonadaceae;__Sphingobium	0.0	0.0	0.0
244	Bacteria;__Proteobacteria;__Betaproteobacteria;__Burkholderiales;__Alcaligenaceae;__Alcaligenes	0.0	0.0	0.0
245	Bacteria;__Proteobacteria;__Betaproteobacteria;__Burkholderiales;__Comamonadaceae;__Pelomonas	0.0	0.1	0.0
246	Bacteria;__Proteobacteria;__Betaproteobacteria;__Burkholderiales;__Oxalobacteraceae;Other	0.0	0.0	0.0
247	Bacteria;__Proteobacteria;__Betaproteobacteria;__Neisseriales;__Neisseriaceae;__Microvirgula	0.0	0.0	0.0
248	Bacteria;__Proteobacteria;__Deltaproteobacteria;__Myxococcales;Other;Other	0.0	0.0	0.0
249	Bacteria;__Proteobacteria;__Deltaproteobacteria;__Myxococcales;__0319-6G20;__g	0.0	0.0	0.0
250	Bacteria;__Proteobacteria;__Epsilonproteobacteria;__Campylobacterales;__Campylobacteraceae;__Arcobacter	0.0	0.0	0.0
251	Bacteria;__Proteobacteria;__Gammaproteobacteria;__Aeromonadales;Other;Other	0.0	0.0	0.0
252	Bacteria;__Proteobacteria;__Gammaproteobacteria;__Aeromonadales;__Aeromonadaceae;__Zobellella	0.0	0.0	0.0
253	Bacteria;__Proteobacteria;__Gammaproteobacteria;__Alteromonadales;Other;Other	0.0	0.0	0.0
254	Bacteria;__Proteobacteria;__Gammaproteobacteria;__Alteromonadales;__Shewanellaceae;__Shewanella	0.0	0.0	0.0
255	Bacteria;__Proteobacteria;__Gammaproteobacteria;__Chromatiales;__Chromatiaceae;__Rheinheimera	0.0	0.0	0.0
256	Bacteria;__Proteobacteria;__Gammaproteobacteria;__Pasteurellales;__Pasteurellaceae;__Haemophilus	0.0	0.0	0.0
257	Bacteria;__Proteobacteria;__Gammaproteobacteria;__Pseudomonadales;__Moraxellaceae;__Moraxella	0.0	0.0	0.0
258	Bacteria;__Spirochaetes;__Spirochaetes;__Spirochaetales;__Spirochaetaceae;__Spirochaeta	0.0	0.0	0.0
259	Bacteria;__Spirochaetes;__Spirochaetes;__Spirochaetales;__Spirochaetaceae;__Treponema	0.0	0.0	0.0
260	Bacteria;__Tenericutes;__Mollicutes;__RF9;__f;__g	0.0	0.0	0.0
261	Unclassified;Other;Other;Other;Other;Other	0.0	0.0	0.0

Average value of the six-times collections.

Figure legends

Figure S1 Prefectures in Japan from which the faecal samples from neonatal calves were collected. Black points indicate the approximate locations of the farm(s). The map was obtained from CraftMAP (http://www.craftmap.box-i.net/) and edited by Microsoft® PowerPoint® 2013.

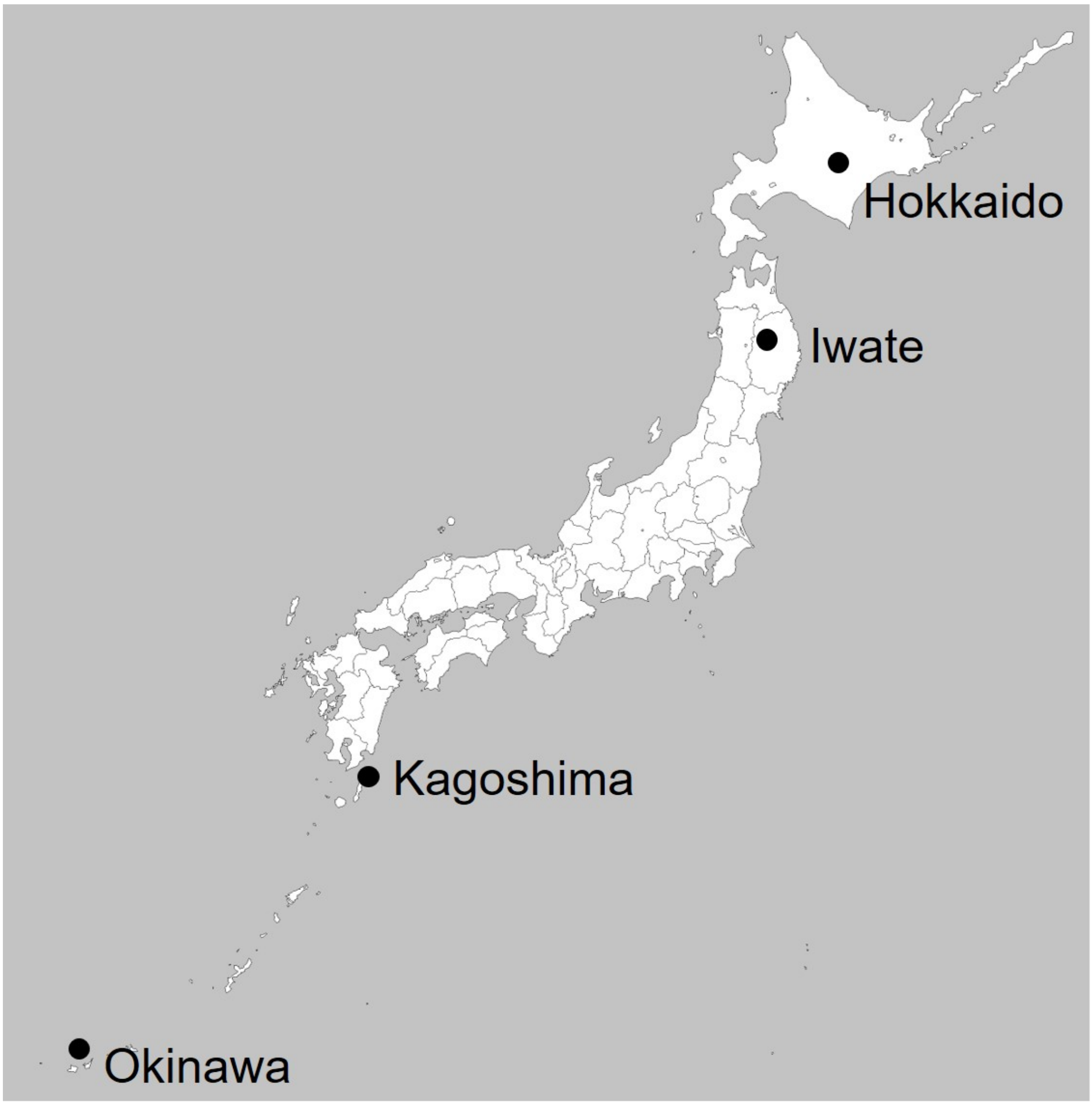

Supplement: Supplementary file 1 — Supplementary material [file 41598_2019_48969_MOESM1_ESM.pdf]
